# *Tisochrysis lutea* F&M-M36 Mitigates Risk Factors of Metabolic Syndrome and Promotes Visceral Fat Browning through β3-Adrenergic Receptor/UCP1 Signaling

**DOI:** 10.3390/md21050303

**Published:** 2023-05-17

**Authors:** Mario D’Ambrosio, Elisabetta Bigagli, Lorenzo Cinci, Manuela Gencarelli, Sofia Chioccioli, Natascia Biondi, Liliana Rodolfi, Alberto Niccolai, Francesca Zambelli, Annunziatina Laurino, Laura Raimondi, Mario R. Tredici, Cristina Luceri

**Affiliations:** 1 Department of NEUROFARBA, Section of Pharmacology and Toxicology, University of Florence, Viale Pieraccini 6, 50139 Florence, Italycristina.luceri@unifi.it (C.L.); 2 Enteric Neuroscience Program, Department of Medicine, Section of Gastroenterology and Hepatology, Mayo Clinic, Rochester, MN 55905, USA; 3 Department of Agriculture, Food, Environment and Forestry (DAGRI), University of Florence, Piazzale delle Cascine 18, 50144 Florence, Italy; 4 Fotosintetica & Microbiologica S.r.l., Via di Santo Spirito 14, 50125 Florence, Italy

**Keywords:** microalgae, *Tisochrysis lutea*, adipose tissue, lipid metabolism, thermogenesis, β3-adrenergic receptor, UCP-1, inflammation, fenofibrate

## Abstract

Pre-metabolic syndrome (pre-MetS) may represent the best transition phase to start treatments aimed at reducing cardiometabolic risk factors of MetS. In this study, we investigated the effects of the marine microalga *Tisochrysis lutea* F&M-M36 (*T. lutea*) on cardiometabolic components of pre-MetS and its underlying mechanisms. Rats were fed a standard (5% fat) or a high-fat diet (20% fat) supplemented or not with 5% of *T. lutea* or fenofibrate (100 mg/Kg) for 3 months. Like fenofibrate, *T. lutea* decreased blood triglycerides (*p* < 0.01) and glucose levels (*p* < 0.01), increased fecal lipid excretion (*p* < 0.05) and adiponectin (*p* < 0.001) without affecting weight gain. Unlike fenofibrate, *T. lutea* did not increase liver weight and steatosis, reduced renal fat (*p* < 0.05), diastolic (*p* < 0.05) and mean arterial pressure (*p* < 0.05). In visceral adipose tissue (VAT), *T. lutea*, but not fenofibrate, increased the β3-adrenergic receptor (β3ADR) (*p* < 0.05) and Uncoupling protein 1 (UCP-1) (*p* < 0.001) while both induced glucagon-like peptide-1 receptor (GLP1R) protein expression (*p* < 0.001) and decreased interleukin (IL)-6 and IL-1β gene expression (*p* < 0.05). Pathway analysis on VAT whole-gene expression profiles showed that *T. lutea* up-regulated energy-metabolism-related genes and down-regulated inflammatory and autophagy pathways. The multitarget activity of *T. lutea* suggests that this microalga could be useful in mitigating risk factors of MetS.

## 1. Introduction

Metabolic syndrome (MetS) is defined as a cluster of metabolic alterations including at least three of the following: abdominal obesity, hypertriglyceridemia, hypertension, low high-density lipoprotein (HDL) cholesterol, and hyperglycemia [1]. Apart from metabolic abnormalities, chronic low-grade inflammation associated with MetS plays a crucial role in increasing cardiovascular diseases and diabetes [2].

The first-line approach in treating MetS relies on improving lifestyle and dietary habits followed by poly-pharmacological strategies able to simultaneously treat individual components of MetS [3]. Another potential approach could be focusing on the pre-disease state, namely pre-metabolic syndrome (pre-MetS), a condition of increased susceptibility to MetS where, however, the diagnostic criteria of MetS are not already met [4]. This pre-disease state could represent the best transition phase to start effective treatments based on dietary interventions.

The marine microalga *Tisochrysis lutea* (*T. lutea*) contains several multitarget bioactive compounds endowed with anti-inflammatory and anti-dyslipidemic properties, such as omega-3 fatty acids, mainly docosahexaenoic acid (DHA), polyphenols, and carotenoids, such as fucoxanthin [5,6,7,8]. Unlike other microalgae, *T. lutea* is not approved for human consumption and it is currently mainly used in aquaculture [9]. We were among the first to evaluate the safety of *T. lutea* F&M-M36 in animals, demonstrating that a diet containing a high percentage (20%) of microalgal biomass was well tolerated in the short term with no remarkable systemic toxic effects or organ damage; however, we also highlighted that the relatively high salt and nucleic acids content of the biomass could limit its inclusion in the diet [5]. In the same study, despite rats being fed a well-balanced and iso-caloric diet, we observed an unexpected, positive effect of *T. lutea* F&M-M36 on lipid metabolism [5]. We also recently demonstrated that the *T. lutea* F&M-M36 methanolic extract exerts anti-inflammatory activity in vitro by reducing the Cyclooxygenases-2 (COX-2) and Prostaglandin E2 (PGE2) pathway and NLRP3 inflammasome/microRNA-223 axis, with effects more evident than those of fucoxanthin alone [6]. In rats fed a high-fat and high-fructose diet, dietary supplementation with 12% *T. lutea* exerted anti-inflammatory effects and ameliorated lipid and glucose metabolism probably in virtue of the potential synergy among DHA, fucoxanthin, phytosterols, and fibers [8]. However, the molecular mechanisms involved, including the genes, proteins, and pathways modulated by the microalga and the analysis of its functional and molecular effects compared to existing drugs have been almost unexplored so far.

In this study, the effects of *T. lutea* F&M-M36 on the main components of pre-MetS, blood lipids, glucose, pressure, and inflammation were investigated; furthermore, since *T. lutea* is rich in DHA [8], a natural ligand of the nuclear receptor peroxisome proliferator-activated receptor α (PPARα), its effects were compared to those of fenofibrate, a synthetic ligand of the same receptor currently used in the pharmacotherapy of dyslipidemia [10]. The underlying mechanisms were also explored focusing on adipose tissue, a culprit organ for the regulation of whole-body energy homeostasis and metabolism.

## 2. Results

### 2.1. Effects of T. lutea F&M-M36 vs. Fenofibrate on Body and Organs Weight and on Fat Mass

Daily food and calorie intakes were significantly higher in rats fed NF diet compared to the other groups (all, *p* < 0.001), whereas water intake was similar. As a result of the lower calorie intake, the high-fat (HF) diet groups did not show a significantly higher weight gain compared to rats fed normal-fat (NF) diet. However, the HF diet increased epididymal and renal fat weight compared to NF diet (*p* < 0.05 and *p* < 0.01, respectively), but not visceral (mesenteric and retroperitoneal) fat. Fenofibrate did not affect fat depots, while *T. lutea* F&M-M36 significantly decreased renal fat weight compared to the HF diet (*p* < 0.05). Rats fed *T. lutea* F&M-M36 and fenofibrate also showed a significantly higher fecal excretion of lipids compared to HF (*p* < 0.05 and *p* < 0.01, respectively) and NF (*p* < 0.05 and *p* < 0.01, respectively). The increased liver and kidney weight in rats treated with fenofibrate (*p* < 0.001) was not observed in those fed *T. lutea* F&M-M36 (Table 1).

### 2.2. Effects of T. lutea F&M-M36 and Fenofibrate on Metabolic Profile, Adiponectin, and Blood Pressure

The HF diet significantly increased plasma triglycerides (*p* < 0.01) compared to NF diet but did not impact total cholesterol or high-density lipoprotein (HDL). *T. lutea* and fenofibrate were both able to significantly reduce TG compared to the HF diet (*p* < 0.01 and *p* < 0.001, respectively) (Table 2). The atherogenic index of plasma (AIP) was also reduced by *T. lutea* supplementation (*p* < 0.05) and fenofibrate (*p* < 0.01). Furthermore, both fenofibrate and *T. lutea* significantly counteracted the increase in plasma non-fasting glucose levels induced by the HF diet (*p* < 0.001 and *p* < 0.01, respectively) (Table 2).

Adiponectin plasma levels were increased by fenofibrate and *T. lutea* compared to the HF diet (both *p* < 0.001); the urinary excretion of uric acid was similar among the groups. Systolic, diastolic, and mean arterial pressures, as well as the rate pressure product were not affected by the HF diet, except for SBP; *T. lutea* F&M-M36 significantly reduced all these parameters (*p* < 0.01, *p* < 0.05 and *p* < 0.05) compared to the HF diet (Table 2).

### 2.3. Effects of T. lutea F&M-M36 and Fenofibrate on Hepatic Steatosis

**Figure 1:**
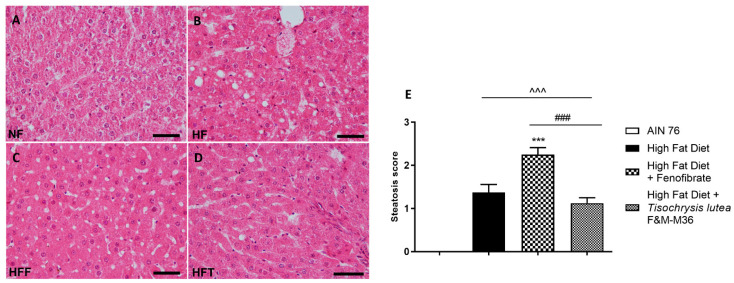
Hematoxylin and eosin staining for histopathological analysis of rat hepatic tissues: (**A**) NF group; (**B**) HF group; (**C**) HFF group; (**D**) HFT group, scale bar 400× magnification; (**E**) steatosis score. Normal diet (NF), high-fat diet (HF), high-fat diet + fenofibrate (HFF), and high-fat diet + *T. lutea* F&M-M36 (HFF). Data are expressed as means ± SE; *n* = 8 rats/group. ^^^ *p* < 0.001 vs. NF; *** *p* < 0.001 vs. HF; ### *p* < 0.001 vs. HFF, by one-way ANOVA and Dunnett’s multiple comparisons test.

Hematoxylin and eosin staining of the liver revealed that the HF diet induced a significant increase in the steatosis score (Figure 1A–E) compared to the NF diet (*p* < 0.001); the score of rats fed *T. lutea* was similar to that of the HF diet (Figure 1D,E); on the contrary, fenofibrate increased the steatosis score compared to the HF diet (*p* < 0.001, Figure 1C,E) and induced hepatocellular ballooning, characterized by hepatocytic diameter enlargement and the presence of lipid droplets (Figure 1C), features of steatotic damage.

### 2.4. Effects of T. lutea F&M-M36 and Fenofibrate on Glycogen Storage in the Liver

**Figure 2:**
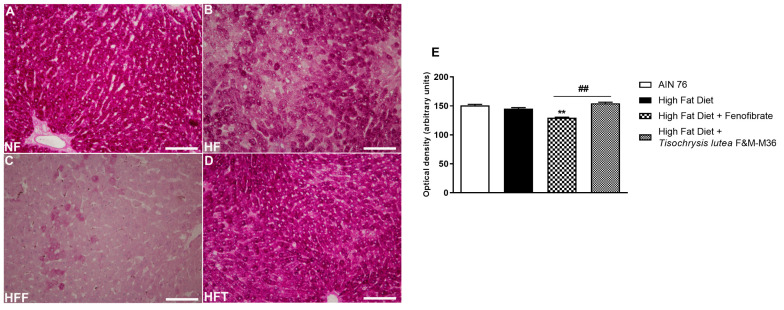
Periodic acid–Schiff (PAS) staining of rat hepatic tissues in the different groups; (**A**) NF group; (**B**) HF group; (**C**) HFF group; (**D**) HFT group. Scale bar 400× magnification. (**E**) glycogen storage. Data are expressed as mean ± SE; *n* = 8 rats/group. ** *p* < 0.01 vs. HF and ## *p* < 0.01 vs. HFF, by one-way ANOVA and Dunnett’s multiple comparisons test.

As shown in Figure 2, the HF diet did not modify glycogen storage determined by periodic acid–Schiff (PAS) glycogen staining, compared to the NF diet. Fenofibrate but not *T. lutea* significantly decreased hepatic glycogen levels, compared to the HF diet (*p* < 0.01).

### 2.5. Effects of T. lutea F&M-M36 and Fenofibrate on β3ADr, Ucp1, and Glp1r Protein Expression in Visceral Adipose Tissue

**Figure 3:**
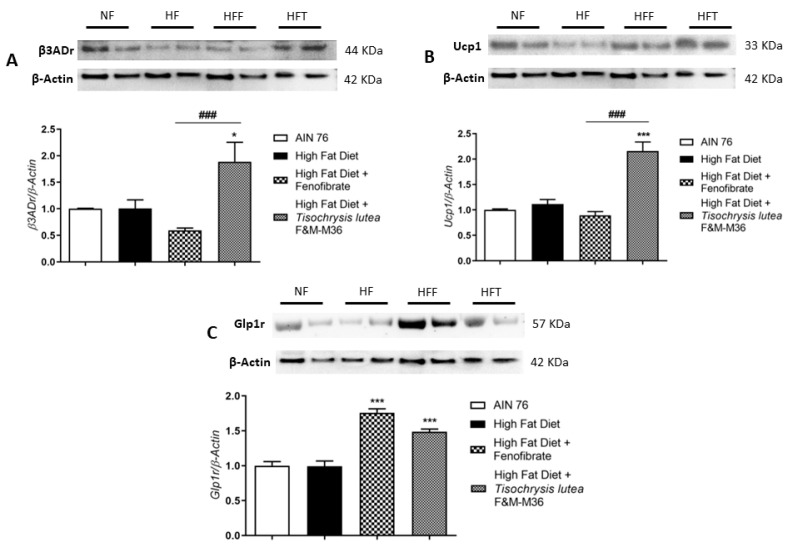
Effect of fenofibrate and *T. lutea* F&M-M36 on type 3 beta adrenergic receptors (β3ADr, (**A**)), Uncoupling Protein 1 (Ucp1, (**B**)) and Glucagon-like peptide-1 receptor (Glp1r, (**C**)) protein expression in visceral adipose tissue by Western-blot analysis. Representative gel images loaded with proteins obtained from two different samples for each experimental group. Normal diet (NF), high-fat diet (HF), high-fat diet + fenofibrate (HFF), and high-fat diet + *T. lutea* F&M-M36 (HFF). Densitometric analysis is expressed as mean ± SE; *n* = 8 rats/group. * *p* < 0.05 and *** *p* < 0.001 vs. HF; ### *p* < 0.001 vs. HFF by one-way ANOVA and Dunnett’s multiple comparisons test.

Western blot analysis on visceral adipose tissue revealed that the HF diet did not affect type 3 beta adrenergic receptor (β3ADr), Uncoupling Protein 1 (Ucp1), and Glucagon-like peptide-1 receptor (Glp1r) protein expression (Figure 3A–C). However, *T. lutea* but not fenofibrate, significantly increased the expression of β3ADr (Panel A, *p* < 0.05) and Ucp1 (Panel B, *p* < 0.001) compared to the HF diet. At a similar extent to fenofibrate, *T. lutea* also significantly induced Glp1r protein expression compared to the HF diet (Panel C, both *p* < 0.001). On the contrary, in brown adipose tissue, the mRNA expression of β3ADr and Ucp1 was similar among groups (data not shown).

### 2.6. Effects of T. lutea F&M-M36 and Fenofibrate on Pro-Inflammatory Cytokines mRNA Expression in Visceral Adipose Tissue

**Figure 4:**
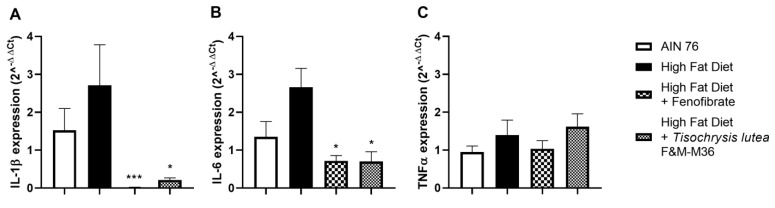
Effect of *T. lutea* F&M-M36 and fenofibrate on interleukin 1β (IL-1β, (**A**)), interleukin 6 (IL-6, (**B**)) and tumor necrosis factor alfa (TNFα, (**C**)) mRNA expression in visceral adipose tissue by real-time PCR. Data are expressed as mean ± SE; *n* = 8 rats/group. * *p* < 0.05 and *** *p* < 0.001 vs. HF by one-way ANOVA and Dunnett’s multiple comparisons test.

As shown in Figure 4, the HF diet slightly induced the mRNA expression of interleukin 1 beta (IL-1β, Figure 4A) interleukin-6 (IL-6, Figure 4B) while not affecting tumor necrosis factor alpha (TNFα, Panel C) compared to the NF diet. Fenofibrate and *T. lutea* F&M-M36 significantly reduced Il-1β (*p* < 0.001 and *p* < 0.05, respectively) and IL-6 (*p* < 0.05 for both) compared to the HF (Figure 4A,B).

### 2.7. Effect of T. lutea F&M-M36 on Whole-Gene Expression Profiles in Visceral Adipose Tissue

Transcriptomic analysis of whole-gene expression in visceral fat identified 2159 differentially expressed genes between HF diet and *T. lutea*-fed rats (*p* < 0.01), out of which 992 were up-regulated and 1167 were down-regulated. Pathway analysis identified 15 KEGG pathways differentially modulated by *T. lutea* compared to the HF diet, 13 up-regulated, and 2 down-regulated (Table 3). Among up-regulated pathways, it is of interest to mention PPAR signaling and Huntington’s, Parkinson’s, and Alzheimer’s disease pathways since they include several electron transport chain and oxidative phosphorylation genes (several ATP synthases (ATPs, cytochrome c oxidases (COXs), and NADH ubiquinone oxidoreductases (Ndufs)) and peroxisome proliferator-activated receptor gamma (PPARγ). Conversely, down-regulated pathways included cytokine–cytokine receptor interaction and regulation of autophagy (Table 3).

## 3. Discussion

High-fat diet is a critical risk factor for the development of obesity and related metabolic disorders [11]. In this study, a high-fat diet (30%) administered to Sprague Dawley rats for 3 months did not induce a frank MetS, but rather a pre-MetS, the initial stage of the disease. There is no consensus on the criteria for defining pre-MetS in humans [12,13], but Gesteiro et al. recently proposed to consider the cardiometabolic risk and the closeness to the cut-off criteria for the diagnosis of MetS [14]. In our model, because of the lower food and calorie intakes, rats fed the HF diet showed a similar weight gain compared to rats fed the normal-fat diet; however, the HF diet had a negative impact on blood lipids and fat depots. Indeed, the HF diet increased renal and epididymal fat deposits, blood triglycerides, and glucose but did not induce obesity or hypertension or a significant increase in visceral adipose tissue, therefore better recapitulating the features of pre-MetS. This stage of the disease can be the ideal timepoint to start dietary interventions aimed at reducing risk factors by acting on the same cardiometabolic routes of overt MetS.

In our previous study on the safety of a diet containing 20% of *T. lutea*, we reported that the relatively high salt and nucleic acid content of the biomass represented a safety concern limiting its inclusion in the diet [5]. Notably, by decreasing the dose to 5%, which is more achievable than dietary consumption, in the present study, we did not observe signs of adverse effects, including histopathological alterations in any organ or abnormalities in water consumption, diuresis or uric acid excretion, indicating that the sub-chronic administration of *T. lutea* is safe in rats, at this dose. On the contrary, Mayer et al. reported a marked increase in water intake in rats fed 12% Tiso with no other relevant abnormalities [8].

This is the first study comparing the effects of a dietary intervention with those of fenofibrate, a widely employed anti-dyslipidemic drug. Its effects, such as those of DHA, are mediated through the activation of PPAR-α, leading to expression of genes involved in lipid metabolism and through reducing lipoprotein lipase [15]. Despite being less effective than fenofibrate, dietary treatment with *T. lutea* reduced plasma triglycerides by nearly 50% without increasing lipid accumulation in the liver; at variance, rats treated with fenofibrate showed enhanced hepatic lipid accumulation, an adverse effect previously reported in ob/ob mice [16]. Non-fasting glucose levels were also decreased by *T. lutea* to the same extent as fenofibrate. These positive effects on lipid and glucose metabolisms agree with the results of Meyer et al., who also reported a significant reduction in body weight, but their animals were fed a high-fat and high-fructose diet, and the dose of *T. lutea* was 12% [8]. Like fenofibrate, *T. lutea* reduced the atherogenic index of plasma, a metabolic syndrome and atherogenicity biomarker [17,18]; *T. lutea* also enhanced adiponectin, an adipocyte-secreted anti-inflammatory protein associated with lower inflammation and with increased antioxidant defenses [19,20].

Interestingly, renal fat accumulation was significantly reduced by *T. lutea* but not by fenofibrate; this result is of relevance since perirenal fat correlates with metabolic risk factors in obese subjects and in patients with chronic kidney disease and type 2 diabetes [21,22,23]. Other than being suggested as an independent predictor of MetS, para-and perirenal fat are also positively correlated with diastolic blood pressure in overweight and obese healthy subjects [24]. Consistently, we observed reduced diastolic blood pressure and renal fat in rats treated with *T. lutea* but not with fenofibrate; mechanistically, the reduction in perirenal fat may limit the compression of renal lymphatics and veins, the renal hydrostatic pressure, and the activation of the renin–angiotensin–aldosterone system [24]. Collectively, these data indicate that *T. lutea* may exert a protective effect against cardiometabolic risk factors of MetS.

Our study is the first to report the molecular mechanisms involved in these effects, focusing on adipose tissue, a key player in energy metabolism. In addition to the well-known role of white adipose tissue (WAT) in storing energy in the form of triglycerides, mammals, including adult humans, are also equipped with brown adipose tissue (BAT) that uses fatty acids and glucose at a high rate and dissipates energy as heat for non-shivering thermogenesis [25]. Adaptive thermogenesis in BAT is principally dependent on the activation of UCP1, a mitochondrial protein specific to brown adipocytes, which uncouples electron transport from ATP production, thereby generating heat [25]. β3-adrenergic receptors are among the predominant regulators of this process and most of the strategies to enhance lipolysis and thermogenesis converge into the stimulation of these receptors [26]. Cold exposure, β3 receptors agonists, but also dietary factors can induce the so-called “WAT browning” process characterized by the presence of UCP1-expressing beige or brown-like adipocytes displaying the same thermogenic function of brown adipocytes [26]. By increasing energy expenditure and regulating glucose and lipid metabolism, “WAT browning” seems a promising approach for MetS and obesity [27].

Our results demonstrate that *T. lutea* increases the expression of the β3-adrenergic receptor and UCP1 in visceral but not in brown fat, suggesting that this microalga stimulates visceral WAT browning and thermogenesis without affecting the metabolic activity of BAT. Various bioactive compounds of *T. lutea* biomass can exert a positive effect on WAT browning; among them, the literature extensively reported that PUFAs, especially ω-3 PUFAs, increase WAT browning and energy expenditure through UCP1-dependent mechanisms. The DHA content in *T. lutea* F&M-M36 is 1.01% of dry matter [5,7], equivalent to an average DHA intake of 13.89 mg/day/rat, and for a 70 kg human, it corresponds to a daily intake of about 2 g, the minimal effective dose to lower plasma triglycerides [28]. *T. lutea* F&M-M36 biomass also contains 0.6% (dry weight) of the carotenoid fucoxanthin [5,7]; fucoxanthin showed anti-obesity and anti-diabetic effects in mice by promoting the expression of the UCP1 and β3-adrenergic receptors in WAT [29,30,31]. In addition, the methanolic extract of *T. lutea* F&M-M36 contains 6.22 mg of gallic acid equivalents of phenols per gram, and in particular, phenolic acid derivatives of hydroxybenzoic and gallic acids [6]. The ability of phenolic compounds to induce WAT browning is largely demonstrated and may involve the activation of β3 signaling pathways [32,33].

Increased fecal lipid excretion was also observed in rats fed *T. lutea* or treated with fenofibrate. Data in mice indicate that fenofibrate decreases cholesterol absorption by reducing the expression of the intestinal cholesterol transporter Niemann-Pick C1-Like 1 (NPC1L1), thereby increasing fecal neutral sterol excretion [34]. Orlistat, another well-known drug able to increase fecal lipid excretion, selectively inhibits gastrointestinal lipase activity, thereby preventing the absorption of dietary fat [35]. The increased lipid excretion observed in *T-lutea*-fed rats may be due to ω-3 and fucoxanthin; indeed, eicosapentaenoic acid (EPA) and DHA limit cholesterol uptake and are transported by down-regulating NPC1L1 [36], while fucoxanthin inhibits gastrointestinal lipase activity and suppresses triglyceride absorption, thereby increasing the fecal excretions of lipids [37,38].

Pathway analysis on adipose tissue whole-gene expression profiles suggested that *T.lutea* increased the adipose tissue metabolic activity. Indeed, among the genes belonging to the most significantly up-regulated pathways, we found multiple energy metabolism-related genes, electron transport chain genes, and those relevant for the control of oxidative phosphorylation and mitochondrial functions, such as *ATP5g*, *Cox4i2*, and *Cox5b* [39,40]. Genes such as *Acox-1*, *FABP4* and *FABP5PPAR*, and *PPARg*, belonging to PPAR signaling, are implicated in thermogenesis, lipolysis promotion, and adipocytes differentiation [40,41,42,43,44,45]. In particular, the overexpression of the mitochondrial *Cox4i2* gene and the β-oxidation *Acox-1* gene was associated with β3-adrenergic receptor activation in adipose tissue [40]. The down-regulation of autophagy is also consistent with the maintenance of fat browning through β-adrenergic stimulation [46].

The inflammation of visceral adipose tissue is also a trigger for the induction and spreading of low-grade systemic inflammation; the down-regulation of the cytokine–cytokine receptor pathway, including several chemokines and interleukins, suggests that *T. lutea* also exerts anti-inflammatory actions in the adipose tissue, thus contributing to the mitigation of chronic low-grade inflammation. It is also intriguing that both fenofibrate and *T. lutea* F&M-M36 induced the expression of GLP1R, down-regulated that of pro-inflammatory cytokines IL-6 and IL-1β in adipose tissue, and increased adiponectin plasma levels, thus suggesting that some of the metabolic effects observed may in part be due to the amplification of incretin action and its known anti-inflammatory effects [47,48].

Collectively, our data suggest that the a role of *T. lutea* F&M-M36 on visceral fat browning is at least in part mediated by the activation of β3ADR/UCP1 signaling in visceral fat, but further studies using appropriate knock-out models are needed to validate this mechanistic pathway.

The ability of *T. lutea* F&M-M36 to target multiple components of pre-MetS suggests that this microalga is a promising candidate for mitigating risk factors associated with metabolic syndrome.

## 4. Materials and Methods

### 4.1. Microalgae Cultivation and Production

*T. lutea* F&M-M36 biomass belongs to the Fotosintetica & Microbiologica (F&M) S.r.l. Culture Collection (Florence, Italy), and was produced at Archimede Ricerche S.r.l. (Camporosso, Imperia, Italy) in GWP^®^-II photobioreactors [49] in a semi-batch mode in F medium [50]. The biomass was harvested by centrifugation, frozen, lyophilized, and powdered. The powdered biomass was stored at −20 °C until use.

### 4.2. Animals and Treatment Design

All procedures were carried out in agreement with the European Union Regulations on the Care and Use of Laboratory Animals (OJ of ECL 358/1, 18 December 1986) and according to Italian regulations on the protection of animals used for experimental and other scientific purposes (DM 116/1992), after approval from the Italian Ministry for Scientific Research (ID 1137/2015; date of approval 28 October 2015).

After 1 week of acclimatization in plastic cages equipped with soft wood bedding, a water bottle, and basic equipment, male Sprague Dawley rats (Nossan S.r.l., Milan, Italy), aged 6–8 weeks, were divided into 4 experimental groups; rats fed AIN-76 diet (NF—5% of fat; *n* = 8), rats fed high-fat diet (HF—30% of fat; *n* = 8), rats fed a HF diet, supplemented with 5% *T. lutea* F&M-M36 biomass (HFT—30% of fat; *n* = 8), and rats fed a HF diet, supplemented with 100 mg/Kg Fenofibrate for 2 months, after the first month of feeding (HFF—30% of fat; *n* = 8).

Water and food were available ad libitum; the lighting regime was a standard 12 h light and 12 h dark regime, the temperature was maintained constant at 21 ± 2 °C. At the end of the study, the rats were euthanized by inhalation of CO_2_, and blood samples were collected in tubes containing an anticoagulant (EDTA) to separate plasma. Following the sacrifice, kidney, hearth, liver, visceral, epididymal, and renal adipose tissue were weighed and stored either at –20 °C or –80 °C for further analyses. The experimental diets were prepared using components purchased from Piccioni Laboratories (Gessate, Milan, Italy). All the diets were prepared in accordance with the standard AIN76 diet. In the HF and HFT diets, the amount of proteins, lipids, carbohydrates, and fibers were adjusted in accordance with the contents of these components in the microalgal biomass (Table 4). The AIN76 diet provided 3.79 kcal/g, while HF and HFT diets provided 5.33 and 5.32 kcal/g, respectively.

### 4.3. Blood Pressure Measurement

Systolic and diastolic blood pressures, mean arterial pressure (MAP), and heart rate (HR) were measured in conscious rats by the noninvasive computerized tail-cuff method (Visitech BP-2000 Series II Blood Pressure Analysis System, Apex, NC, USA). Before the recording, 2 days of training were performed for each animal. The same researcher performed all measurements, repeated 10 times, in a quiet environment; the highest and lowest values were discarded. The values recorded in the rats of each experimental group were reported as mean ± SEM. The rate pressure product (RPP), which is an indirect index of myocardial oxygen consumption, was calculated in accordance with [51].

### 4.4. Food and Water Consumption

During the third month of treatment, the animals were placed in metabolic cages for one day to accurately measure water and food daily consumption and the 24 h urine and feces production. Daily calorie intake was calculated by multiplying the daily food intake (g) by the total energy of the diet (kcal/g).

### 4.5. Macroscopic Examinations and Histological Analyses

A complete necropsy, including the examination of the external surface of all orifices and of the cranial, thoracic, abdominal, and pelvic cavities and viscera, was conducted on all rats. The main organs (heart, kidneys, liver, and fat) were weighed. For histological analyses, tissue samples were stored in neutral formaldehyde and embedded in paraffin wax. Sections (5 µm) were stained with a standard hematoxylin and eosin procedure.

Microscopic analysis was performed using the ACT-2U software (Nikon, Instruments Europe, Badhoevedorp, The Netherlands) connected via a camera to the microscope (Optiphot-2; Nikon). Hepatic fat content was evaluated by quantifying the extent of fat droplets in each liver section using the ImageJ software (ImageJ 1.33 image analysis software (https://rsb.info.nih.gov/ij, accessed on 1 May 2023) and expressed as the mean number of lipid droplets for microscopic field. The steatosis score was graded based on the percentage of lipid droplets within the hepatocytes: Grade 0 (healthy, <5%), Grade 1 (mild, 5–33%), Grade 2 (moderate, 34–66%), and Grade 3 (severe, >66%) [52].

### 4.6. Blood Biochemistry

Plasma levels of TC, HDL, Triglycerides, and Glucose were measured with the Reflotron^®^ Plus system (Roche Diagnostics GmbH, Mannheim, Germany) using specific test strips. The atherogenic risk was evaluated by calculating the atherogenic index plasma (AIP) as the log (TAG/HDL-C) [53].

### 4.7. Fecal Lipid Content

Fecal lipid content was measured from dried fecal samples (about 30 mg) in accordance with [54]. Briefly, dried fecal samples (about 30 mg) were re-suspended in 500 μL of normal saline. Then, 500 μL of chloroform–methanol (2:1, *v*/*v*) were added to extract the lipids.

### 4.8. Periodic Acid–Schiff (PAS) Staining

The detection of glycogen in liver tissue was performed on histological slices (4 µm tick). Briefly, tissues were oxidized in 0.5% periodic acid solution (0.5 g of periodic acid in 100 mL distilled water) for 5 min. Washed in distilled water; placed in Schiff reagent (bis-N-aminosulfonic acid) for 15 min; washed in lukewarm tap water for 5 min; counterstained in Mayer’s hematoxylin for 1 min; washed in tap water for 5 min. Microscopic images were evaluated using the ACT-2U software program (Nikon, Instruments Europe, Badhoevedorp, The Netherlands) connected via a camera to the microscope (Optiphot-2; Nikon).

### 4.9. Western Blot

For Western Blot analysis, protein extraction was performed on 50 mg of visceral adipose tissue in accordance with [55]. The total protein content was estimated by using the Bio-Rad DC protein assay kit (Bio-Rad, Segrate, Milan, Italy), using a bovine serum albumin (BSA) standard solution (ranging from 0.2 to 2 mg/mL) for the calibration curve.

Twenty micrograms of proteins were separated on 4–20% SDS-PAGE (Thermo Fisher scientific, Waltham, MA, USA) and transferred into PVDF membranes (60 min at 398 mA) using standard procedures. Blots were incubated overnight at 4 °C with a specific primary antibody (Table 5) diluted in phosphate-buffered saline (PBS) containing 5% BSA or 5% non-fat dry milk and 0.05% Tween 20. The antigen–antibody complexes were visualized using appropriate secondary antibodies (1:10,000, diluted in PBS containing 5% albumin or 5% non-fat dry milk and 0.05% Tween 20), and left for 1 h at room temperature. Blots were then extensively washed with PBS containing 0.1% Tween 20 and developed using an enhanced chemiluminescence detection system (Pierce, Rodano, Italy). Exposition and developing time were standardized for all blots. Densitometric analysis was performed using the public domain NIH Image program (Image J software Version 1.50i, National Institute of Health, Bethesda, MD, USA). Each gel was loaded with all the experimental groups to standardize the image acquisition and densitometric analysis. Data are presented as mean ± SEM of four different gel preparations and were reported as arbitrary units (AU), consisting of the ratio between the level of the target protein expression and that of the β-Actin.

### 4.10. Total RNA Extraction and Real-Time PCR

Total RNA was extracted by using TRIzol extraction reagent (Invitrogen, Carlsbad, CA, USA). First-strand cDNA synthesis was performed using the Revert Aid RT Kit, in accordance with manufacturer’s instruction (Thermo Scientific, Waltham, USA. qRT-PCR assays were carried out in Rotor-Gene^®^qPCR System (Qiagen, Hilden, Germany), using SsoAdvanced Universal SYBR Green Supermix (Biorad, Hercules, CA, USA). Briefly, each reaction was performed in a final volume of 10 μL containing 1 μL of the cDNA, 1 μL of forward and 1 μL of reverse primers, 5 μL of SsoAdvanced universal SYBR Green supermix and 1 μL of nuclease-free water. Primers were designed based on the mouse GenBank sequences for IL-6, IL-1β, and TNFa, and are reported in Table 6. The amplification protocol was based on an initial heat activation at 95 °C for 30 s, followed by 35 cycles of denaturation at 95 °C for 15 s and combined annealing/extension at 60 °C for 30 s. The relative expression of mRNA was normalized by β-Actin and calculated by the 2^−ΔΔCt^ method.

### 4.11. Gene Expression Profiling

Total RNA was extracted from visceral fat by using the RNeasy Mini kit Plus (Macherey-Nagel), in accordance with the manufacturer’s protocol. RNA concentration and purity was determined by using a NanoPhotometer spectrophotometer (IMPLEN, München, Germany).

Transcriptomic analyses were performed using a two-color microarray protocol, in which a pool of RNA (50 ng) extracted from the HFT group (*n* = 6) was contrasted with a reference RNA (50 ng) obtained by pooling an equal amount of RNA samples extracted from HF rats (*n* = 6).

RNA samples were labeled using the Agilent Quick Amp Labeling Kit (Agilent Technologies, Santa Clara, CA, USA), following the manufacturer’s protocol (Two-Color Microarray-Based Gene Expression Analysis Low Input Quick Amp Labeling Version 6.9.1). The RNA Spike-In kit (Agilent Technologies) was also used to monitor and calibrate the linearity, sensitivity, and accuracy of the microarray workflow. Yields of cRNA and the dye incorporation rate were measured with the NanoPhotometer spectrophotometer (IMPLEN). Sample mixture was loaded into the Agilent Rat GE 8 × 60 K v2 Oligo 60-mer microarrays, in Agilent microarray chambers (G2534A) at 65° for 18 h. Fluorescent signal intensities were detected using the Agilent Scan Control 7.0 Software on an Agilent DNA Microarray Scanner, at a resolution of 2 µm. Data were acquired using the Agilent Feature Extraction 9.5.3.1 software. Image analysis and initial quality control were performed using the same software. The genes of interest were those that exhibited a PValueLogRatio (statistical significance on the LogRatio per each gene between red and green channels) of <0.01.

Pathway analysis was performed by means of GO-Elite software, version 1.2.5, an open source, freely available (http://www.genmapp.org/go_elite accessed on 17 January 2023), using the list of the differentially expressed genes with a statistical significance of less than 0.01 and a fold change (FC) of ≥2 or ≤2 as input data.

### 4.12. Statistics

Statistical analyses were conducted using the GraphPad Prism 8.02 software (GraphPad, San Diego, CA, USA) program. The D’Agostino and Pearson omnibus normality test was applied to verify the Gaussian distribution of each variable. Differences among groups were analyzed by using one-way ANOVA and Dunnett’s multiple comparisons test or by Kruskal–Wallis and Dunn’s multiple comparisons tests, when appropriate. Results are presented as means ± SEM. Significance was assigned at *p* < 0.05.

## Figures and Tables

**Table 1:** Effects of *T. lutea* F&M-M36 and fenofibrate on body weight gain, fat weight, food, and water intake.

	NF	HF	HFF	HFT
Weight gain (g)	213.5 ± 7.7	252.5 ± 20.1	208.5 ± 11.6	281.3 ± 11.6
Food intake (g)	51.66 ± 0.3	27.51 ± 0.5 ^^^	31.55 ± 0.5 ^^^	27.51 ± 0.5 ^^^
Calorie intake (kcal)/24 h	195.8 ± 1.3	146.6 ± 2.64 ^^^	168.2 ± 2.4 ^^^	146.3 ± 2.63 ^^^
Water intake (mL)	52.47 ± 2	50.27 ± 1.8	58.27 ± 1.7	52.80 ± 1.2
Fecal lipids excretion (µg/g 24 h dw)	1.4 ± 0.2	2.0 ± 0.2	5.4 ± 1.2 ^^**	4.2 ± 0.6 ^*
Liver (w/bw × 10^−3^)	38.86 ± 0.80	36.85 ± 1.38	65.44 ± 0.95 ***^^^	32.63 ± 0.29
Kidney (w/bw × 10^−3^)	3.23 ± 0.07	3.18 ± 0.10	4.21 ± 0.13 ***^^^	3.04 ± 0.10
Heart (w/bw × 10^−3^)	3.53 ± 0.12	3.38 ± 0.06	3.41 ± 0.09	3.70 ± 0.09
Visceral fat (w/bw × 10^−3^)	1.38 ± 0.14	2.30 ± 0.24	2.04 ± 0.39	1.83 ± 0.22
Epididymal fat (w/bw × 10^−3^)	7.64 ± 0.73	12.37 ± 0.67 ^	10.24 ± 1.61	10.93 ± 1.20
Renal fat (w/bw × 10^−3^)	8.56 ± 1.40	15.89 ± 1.49 ^^	12.46 ± 1.40	10.26 ± 0.55 *

Normal diet (NF), high-fat diet (HF), high-fat diet + fenofibrate (HFF), high-fat diet + *T. lutea* F&M-M36 (HFT), dry weight (dw), ratio of organ weights to body weight (w/bw). Water and food intakes and fecal lipids excretion were evaluated during the third month of treatment, placing rats in metabolic cages for one day. Data are expressed as means ± SE; *n* = 8 rats/group. ^ *p* < 0.05, ^^ *p* < 0.01 and ^^^ *p* < 0.001 vs. NF; * *p* < 0.05, ** *p* < 0.01 and *** *p* < 0.001 vs. HF, by one-way ANOVA and Dunnett’s multiple comparisons test.

**Table 2:** Effects of *T. lutea* F&M-M36 and fenofibrate on plasma lipids, glucose, adiponectin, atherogenic index, uric acid, and blood pressure.

	NF	HF	HFF	HFT
TG (mg/dL)	183.7 ± 15.1	254.0 ± 21.6 ^^	90.5 ± 3.2 ***^^^	141.0 ± 7.9 **
TC (mg/dL)	128 ± 5.1	134 ± 7.0	130 ± 8.3	139 ± 22.2
HDL (mg/dL)	89 ± 5.64	68 ± 7.8	78 ± 6.5	80 ± 10.2
AIP	2.2 ± 0.36	3.5 ± 0.5	1.2 ± 0.1 **	2.0 ± 0.36 *
Glucose (mg/dL)	168.2 ± 9.1	212.1 ± 11.1 ^	137.5 ± 9.4 ***	150.9 ± 9.1 **
Adiponectin (ng/mL)	37.9 ± 3.5	28.9 ± 1.8	80.5 ± 5.9 ***^^^	57.9 ± 3.4 ***^^
Urinary uric acid (mg/dL)	21.07 ± 4.6	25.05 ± 7.5	14.80 ± 6.1	13 ± 3.9
SBP (mm Hg)	156.2 ± 5.2	159.7 ± 3.6	164.6 ± 2.8	147.9 ± 1.9
DBP (mm Hg)	94.9 ± 6.4	111.7 ± 4.4	105.9 ± 8.1	80.56 ± 4.4 **
MAP (mm Hg)	115.3 ± 5.6	127.7 ± 4.1	125.4 ± 5.5	107.1 ± 4.9 *
RPP (mm Hg bpm)	61,342 ± 2650	68,703 ± 3012	67,409 ± 3093	58,338 ± 1555 *

Normal diet (NF), high-fat diet (HF), high-fat diet + fenofibrate (HFF), and high-fat diet + *T. lutea* F&M-M36 (HFT). Triglycerides (TG), total cholesterol (TC), high density lipoprotein (HDL), atherogenic index of plasma (AIP), SBP (systolic blood pressure), DBP (diastolic blood pressure), MAP (mean arterial pressure), and RPP (rate pressure product). Data are expressed as means ± SE; *n* = 8 rats/group. ^ *p* < 0.05, ^^ *p* < 0.01 and ^^^ *p* < 0.001 vs. NF; * *p* < 0.05, ** *p* < 0.01 and *** *p* < 0.001 vs. HF by one-way ANOVA and Dunnett’s multiple comparisons test.

**Table 3:** List of KEGG gene sets differentially modulated by comparing visceral adipose tissue from high-fat diet and high-fat diet + *T. lutea* F&M-M36, divided into 13 up-regulated and 2 down-regulated.

Up-Regulated			
Gene-Set Name	Percent Changed	PermuteP	Gene Symbols
Notch signaling pathway:KEGG-rno04330	50.0	0.002	Dll3|Dtx1|Jag2|Ncstn|Notch3|Notch4|Ptcra|Rbpj
Protein export:KEGG-rno03060	44.4	0.04	LOC100361694|Oxa1l|Sec11c|Spcs2
Mismatch repair:KEGG-rno03430	44.4	0.05	Exo1|Lig1|Mlh1|Pold3
Galactose metabolism:KEGG-rno00052	41.7	0.03	Gaa|Galk1|Hk2|Hk3|Pfkl
Endocrine and other factor-regulated calcium reabsorption:KEGG-rno04961	33.3	0.02	Ap2b1|Ap2m1|Clta|Cltb|Cltc|Dnm2|Plcb3|Plcb4|Prkcg
PPAR signaling pathway:KEGG-rno03320	33.3	0.01	Acox1|Acsbg1|Dbi|Fabp4|Fabp5|LOC681458|Mmp1|Nr1h3|Pparg|Scd1|Slc27a1|Ubc
Huntington’s disease:KEGG-rno05016	31.9	0.00	Ap2b1|Ap2m1|Atp5g1|Atp5g2|Atp5g3|Atp5hl1|Atp5o|Clta|Cltb|Cltc|Cox4i2|Cox5b|Dnali1|Grm5|LOC688963|Mt-co1|Ndufa10|Ndufa6|Ndufb10|Ndufb11|Ndufb2|Ndufs7|Ndufv1|Plcb3|Plcb4|Pparg|Sod1|Tp53|Uqcrh|Vdac3
Gap junction:KEGG-rno04540	31.3	0.02	Adcy5|Gnai2|Grm5|Htr2a|Plcb3|Plcb4|Prkcg|Tuba3a|Tubb4b|Tubb5
Bacterial invasion of epithelial cells:KEGG-rno05100	31.3	0.02	Arpc1a|Cdc42|Clta|Cltb|Cltc|Ctnnb1|Cttn|Dnm2|Pik3r2|Pxn
Pancreatic secretion:KEGG-rno04972	30.8	0.03	Adcy5|Atp2a3|Cela2a|Clca1|Cpa1|Ctrb1|Pla2g1b|Plcb3|Plcb4|Prkcg|Rap1b|Slc4a2
Antigen processing and presentation:KEGG-rno04612	28.9	0.04	Calr|Ctsb|Hsp90aa1|LOC680121|LOC688090|Psme2|RT1-CE3|RT1-Da|RT1-M1-4|RT1-M6-2|RT1-T18
Parkinson’s disease:KEGG-rno05012	28.4	0.004	Atp5g1|Atp5g2|Atp5g3|Atp5hl1|Atp5o|Cox4i2|Cox5b|Gp1bb|LOC688963|Mt-co1|Ndufa10|Ndufa6|Ndufb10|Ndufb11|Ndufb2|Ndufs7|Ndufv1|Th|Uba1|Ubc|Ube2l3|Uqcrh|Vdac3
Alzheimer’s disease:KEGG-rno05010	25.0	0.03	Atp2a3|Atp5g1|Atp5g2|Atp5g3|Atp5hl1|Atp5o|Calm1|Cox4i2|Cox5b|Grin2c|LOC688963|Mt-co1|Ncstn|Ndufa10|Ndufa6|Ndufb10|Ndufb11|Ndufb2|Ndufs7|Ndufv1|Plcb3|Plcb4|Uqcrh
*Down-regulated*			
*Gene-Set Name*	*Percent Changed*	*PermuteP*	*gene symbols*
Cytokine-cytokine receptor interaction:KEGG-rno04060	26.0	0.0085	Ccl12|Ccr6|Cd40|Csf2rb|Cxcl11|Cxcl13|Cxcr4|Egf|Ifna2|Ifnar2|Il11ra1|Il12b|Il22|LOC100910178|Lta|Osmr|Pdgfrb|RGD1561246|Tnfrsf12a|Tnfrsf21|Tnfrsf8
Regulation of autophagy:KEGG-rno04140	40	0.049	Becn1|Ifna2|Prkaa2|RGD1561246

**Table 4:** Composition of the experimental diets (g/100 g of diet).

	AIN-76 Diet (NF)	High-Fat Diet (HF)	*T. lutea* F&M-M36 Enriched Diet (HFT)
Lyophilized algal biomass			5
Corn oil	5	3	**2**
Lard	-	30	30
Sucrose	50	34	**33.4**
Starch	15		
Casein	20	24.6	**22.5**
Cellulose	5	2	**1.1**
Mineral Mix AIN 76	3.5	4.1	4.1
Vitamin Mix AIN 76	1	1.3	1
Coline	0.2	0.26	0.26
DL Methionine	0.3	0.4	0.4

Values in bold indicate constituents of the diets that were adjusted to compensate for components deriving from algal biomass.

**Table 5:** Primary antibodies used for Western blot analysis (WB).

Antibody	Dilution	Supplier
β3ADr	1:500	Santa Cruz Biotechnology Inc. Dallas, TX, USA (SC-515763)
UCP-1	1:500	Santa Cruz Biotechnology Inc. Dallas, TX, USA (SC-2934184)
GLP1r	1:200	Santa Cruz Biotechnology Inc. Dallas, TX, USA (sc-390774)
β-Actin	1:1000	Bioss Antibodies Woburn, MS, USA (bs-0061R)

**Table 6:** Primer sequences used for real-time PCR.

Gene	Primer Forward	Primer Reverse
β-Actin	TACAGCTTCACCACCACAGC	TGGCCATCTCTTGCTCGAAG
IL-1β	GACTTCACCATGGAACCCGT	GGAGACTGCCCATTCTCGAC
IL-6	GTGGCTAAGGACCAAGACCA	TAGCACACTAGGTTTGCCGAG
TNFα	AACACACGAGACGCTGAAGT	TCCAGTGAGTTCCGAAAGCC
ADRB3	ACTCACCGCTCAACAGGTTT	TTCTGGAGAGTTGCGGTTCC
UCP1	CCGAAACTGTACAGCGGTCT	CAGGAGTGTGGTGCAAAACC

## Data Availability

The data that support the findings of this study are available on request from the corresponding author E.B. (elisabetta.bigagli@unifi.it).

## References

[1] Huang P.L. (2009). A comprehensive definition for metabolic syndrome. Dis. Model Mech..

[2] Hotamisligil G. (2006). Inflammation and metabolic disorders. Nature.

[3] Grundy S.M. (2006). Drug therapy of the metabolic syndrome: Minimizing the emerging crisis in polypharmacy. Nat. Rev. Drug Discov..

[4] Koizumi K., Oku M., Hayashi S., Inujima A., Shibahara N., Chen L., Igarashi Y., Tobe K., Saito S., Kadowaki M. (2019). Identifying pre-disease signals before metabolic syndrome in mice by dynamical network biomarkers. Sci. Rep..

[5] Bigagli E., Cinci L., Niccolai A., Biondi N., Rodolfi L., D’Ottavio M., D’Ambrosio M., Lodovici M., Tredici M.R., Luceri C. (2018). Preliminary data on the dietary safety, tolerability and effects on lipid metabolism of the marine microalga *Tisochrysis lutea*. Algal. Res..

[6] Bigagli E., D’Ambrosio M., Cinci L., Niccolai A., Biondi N., Rodolfi L., Dos Santos Nascimiento L.B., Tredici M.R., Luceri C. (2021). A Comparative In Vitro Evaluation of the Anti-Inflammatory Effects of a *Tisochrysis lutea* Extract and Fucoxanthin. Mar. Drugs.

[7] Niccolai A., Chini Zittelli G., Rodolfi L., Biondi N., Tredici M.R. (2019). Microalgae of interest as food source: Biochemical composition and digestibility. Algal Res..

[8] Mayer C., Richard L., Côme M., Ulmann L., Nazih H., Chénais B., Ouguerram K., Mimouni V. (2021). The Marine Microalga, *Tisochrysis lutea*, Protects against Metabolic Disorders Associated with Metabolic Syndrome and Obesity. Nutrients.

[9] Custódio L., Soares F., Pereira H., Barreira L., Vizetto-Duarte C., Rodrigues M.J., Rauter A.P., Alberício F., Varela J. (2014). Fatty acid composition and biological activities of Isochrysis galbana T-ISO, *Tetraselmis* sp. and *Scenedesmus* sp.: Possible application in the pharmaceutical and functional food industries. J. Appl. Phycol..

[10] Tenenbaum A., Fisman E.Z. (2012). Fibrates are an essential part of modern anti-dyslipidemic arsenal: Spotlight on atherogenic dyslipidemia and residual risk reduction. Cardiovasc. Diabetol..

[11] Duan Y., Zeng L., Zheng C., Song B., Li F., Kong X., Xu K. (2018). Inflammatory Links Between High Fat Diets and Diseases. Front. Immunol..

[12] Vidigal Fde C., Ribeiro A.Q., Babio N., Salas-Salvadó J., Bressan J. (2015). Prevalence of metabolic syndrome and pre-metabolic syndrome in health professionals: Latinmets brazil study. Diabetol. Metab. Syndr..

[13] de las Fuentes L., Brown A.L., Mathews S.J., Waggoner A.D., Soto P.F., Gropler R.J., Dávila-Román V.G. (2007). Metabolic syndrome is associated with abnormal left ventricular diastolic function independent of left ventricular mass. Eur. Heart J..

[14] Gesteiro E., Megía A., Guadalupe-Grau A., Fernandez-Veledo S., Vendrell J., González-Gross M. (2021). Early identification of metabolic syndrome risk: A review of reviews and proposal for defining pre-metabolic syndrome status. Nutr. Metab. Cardiovasc. Dis..

[15] Ferreira A.V., Parreira G.G., Green A., Botion L.M. (2006). Effects of fenofibrate on lipid metabolism in adipose tissue of rats. Metabolism.

[16] Zhang Y., Jia X.B., Liu Y.C., Yu W.Q., Si Y.H., Guo S.D. (2022). Fenofibrate enhances lipid deposition via modulating PPARγ, SREBP-1c, and gut microbiota in ob/ob mice fed a high-fat diet. Front. Nutr..

[17] Zhang X., Zhang X., Li X., Feng J., Chen X. (2019). Association of metabolic syndrome with atherogenic index of plasma in an urban Chinese population: A 15-year prospective study. Nutr. Metab. Cardiovasc. Dis..

[18] Wu J., Zhou Q., Wei Z., Wei J., Cui M. (2021). Atherogenic Index of Plasma and Coronary Artery Disease in the Adult Population: A Meta-Analysis. Front. Cardiovasc. Med..

[19] Rosenson R.S. (2009). Effect of fenofibrate on adiponectin and inflammatory biomarkers in metabolic syndrome patients. Obes. Silver Spring.

[20] Lodovici M., Bigagli E., Tarantini F., Di Serio C., Raimondi L. (2015). Losartan reduces oxidative damage to renal DNA and conserves plasma antioxidant capacity in diabetic rats. Exp. Biol. Med..

[21] Manno C., Campobasso N., Nardecchia A., Triggiani V., Zupo R., Gesualdo L., Silvestris F., De Pergola G. (2019). Relationship of para- and perirenal fat and epicardial fat with metabolic parameters in overweight and obese subjects. Eat Weight Disord..

[22] D’Marco L., Salazar J., Cortez M., Salazar M., Wettel M., Lima-Martínez M., Rojas E., Roque W., Bermúdez V. (2019). Perirenal fat thickness is associated with metabolic risk factors in patients with chronic kidney disease. Kidney Res. Clin. Pract..

[23] Guo X.L., Tu M., Chen Y., Wang W. (2022). Perirenal Fat Thickness: A Surrogate Marker for Metabolic Syndrome in Chinese Newly Diagnosed Type 2 Diabetes. Front. Endocrinol..

[24] De Pergola G., Campobasso N., Nardecchia A., Triggiani V., Caccavo D., Gesualdo L., Silvestris F., Manno C. (2015). Para- and perirenal ultrasonographic fat thickness is associated with 24-h mean diastolic blood pressure levels in overweight and obese subjects. BMC Cardiovasc. Disord..

[25] El Hadi H., Di Vincenzo A., Vettor R., Rossato M. (2019). Food Ingredients Involved in White-to-Brown Adipose Tissue Conversion and in Calorie Burning. Front. Physiol..

[26] Tabuchi C., Sul H.S. (2021). Signaling Pathways Regulating Thermogenesis. Front. Endocrinol..

[27] Ricquier D. (2011). Uncoupling protein 1 of brown adipocytes, the only uncoupler: A historical perspective. Front. Endocrinol..

[28] Park Y., Harris W.S. (2009). Dose-dependent effects of n-3 polyunsaturated fatty acids on platelet activation in mildly hypertriglyceridemic subjects. J. Med. Food.

[29] Maeda H., Hosokawa M., Sashima T., Murakami-Funayama K., Miyashita K. (2009). Anti-obesity and anti-diabetic effects of fucoxanthin on diet-induced obesity conditions in a murine model. Mol. Med. Rep..

[30] Maeda H., Hosokawa M., Sashima T., Funayama K., Miyashita K. (2005). Fucoxanthin from edible seaweed, Undaria pinnatifida, shows antiobesity effect through UCP1 expression in white adipose tissues. Biochem. Biophys. Res. Commun..

[31] Wu M.T., Chou H.N., Huang C.J. (2014). Dietary fucoxanthin increases metabolic rate and upregulated mRNA expressions of the PGC-1alpha network, mitochondrial biogenesis and fusion genes in white adipose tissues of mice. Mar. Drugs.

[32] Hu J., Wang Z., Tan B.K., Christian M. (2020). Dietary polyphenols turn fat “brown”: A narrative review of the possible mechanisms. Trends Food Sci. Technol..

[33] Kang N.H., Mukherjee S., Yun J.W. (2019). Trans-Cinnamic Acid Stimulates White Fat Browning and Activates Brown Adipocytes. Nutrients.

[34] Valasek M.A., Clarke S.L., Repa J.J. (2007). Fenofibrate reduces intestinal cholesterol absorption via PPARalpha-dependent modulation of NPC1L1 expression in mouse. J. Lipid Res..

[35] Johnson S., Schwartz S.M. (2018). Pharmacologic and Pharmacodynamic Equivalence of 2 Formulations of Orlistat. Clin. Pharmacol. Drug Dev..

[36] Yang F., Chen G., Ma M., Qiu N., Zhu L., Li J. (2018). Fatty acids modulate the expression levels of key proteins for cholesterol absorption in Caco-2 monolayer. Lipids Health Dis..

[37] Matsumoto M., Hosokawa M., Matsukawa N., Hagio M., Shinoki A., Nishimukai M., Miyashita K., Yajima T., Hara H. (2010). Suppressive effects of the marine carotenoids, fucoxanthin and fucoxanthinol on triglyceride absorption in lymph duct-cannulated rats. Eur. J. Nutr..

[38] Ha A.W., Kim W.K. (2013). The effect of fucoxanthin rich power on the lipid metabolism in rats with a high fat diet. Nutr. Res. Pract..

[39] Wei X.H., Guo X., Pan C.S., Li H., Cui Y.C., Yan L., Fan J.Y., Deng J.N., Hu B.H., Chang X. (2021). Quantitative Proteomics Reveal That Metabolic Improvement Contributes to the Cardioprotective Effect of T89 on Isoproterenol-Induced Cardiac Injury. Front. Physiol..

[40] Suárez J., Rivera P., Arrabal S., Crespillo A., Serrano A., Baixeras E., Pavón F.J., Cifuentes M., Nogueiras R., Ballesteros J. (2014). Oleoylethanolamide enhances β-adrenergic-mediated thermogenesis and white-to-brown adipocyte phenotype in epididymal white adipose tissue in rat. Dis. Model Mech..

[41] Dou H.X., Wang T., Su H.X., Gao D.D., Xu Y.C., Li Y.X., Wang H.Y. (2020). Exogenous FABP4 interferes with differentiation, promotes lipolysis and inflammation in adipocytes. Endocrine.

[42] Mita T., Furuhashi M., Hiramitsu S., Ishii J., Hoshina K., Ishimura S., Fuseya T., Watanabe Y., Tanaka M., Ohno K. (2015). FABP4 is secreted from adipocytes by adenyl cyclase-PKA- and guanylyl cyclase-PKG-dependent lipolytic mechanisms. Obes. Silver Spring.

[43] Senga S., Kobayashi N., Kawaguchi K., Ando A., Fujii H. (2018). Fatty acid-binding protein 5 (FABP5) promotes lipolysis of lipid droplets, de novo fatty acid (FA) synthesis and activation of nuclear factor-kappa B (NF-κB) signaling in cancer cells. Biochim. Biophys. Acta Mol. Cell Biol. Lipids..

[44] Jeong Y.S., Hong J.H., Cho K.H., Jung H.K. (2012). Grape skin extract reduces adipogenesis- and lipogenesis-related gene expression in 3T3-L1 adipocytes through the peroxisome proliferator-activated receptor-γ signaling pathway. Nutr. Res..

[45] Chen K., Wang L., Yang W., Wang C., Hu G., Mo Z. (2017). Profiling of differentially expressed genes in adipose tissues of multiple symmetric lipomatosis. Mol. Med. Rep..

[46] Ferhat M., Funai K., Boudina S. (2019). Autophagy in Adipose Tissue Physiology and Pathophysiology. Antioxid. Redox Signal..

[47] Lee Y.S., Park M.S., Choung J.S., Kim S.S., Oh H.H., Choi C.S., Ha S.Y., Kang Y., Kim Y., Jun H.S. (2012). Glucagon-like peptide-1 inhibits adipose tissue macrophage infiltration and inflammation in an obese mouse model of diabetes. Diabetologia.

[48] Izaguirre M., Gómez-Ambrosi J., Rodríguez A., Ramírez B., Becerril S., Valentí V., Moncada R., Unamuno X., Silva C., de la Higuera M. (2019). GLP-1 Limits Adipocyte Inflammation and Its Low Circulating Pre-Operative Concentrations Predict Worse Type 2 Diabetes Remission after Bariatric Surgery in Obese Patients. J. Clin. Med..

[49] Tredici M.R., Rodolfi L., Biondi N., Bassi N., Sampietro G. (2016). Techno-economic analysis of microalgal biomass production in a 1-ha Green Wall Panel (GWP^®^) plant. Algal Res..

[50] Guillard R.R.L., Ryther J.H. (1962). Studies of marine planktonic diatoms. I. Cyclotella nana Hustedt and Detonula confervacea Cleve. Can. J. Microbiol..

[51] Whitman M., Jenkins C. (2021). Rate pressure product, age predicted maximum heart rate or heart rate reserve. Which one better predicts cardiovascular events following exercise stress echocardiography?. Am. J. Cardiovasc. Dis..

[52] Takahashi Y., Fukusato T. (2014). Histopathology of nonalcoholic fatty liver disease/nonalcoholic steatohepatitis. World J. Gastroenterol..

[53] Shen S.W., Lu Y., Li F., Yang C.J., Feng Y.B., Li H.W., Yao W.F., Shen Z.H. (2018). Atherogenic index of plasma is an effective index for estimating abdominal obesity. Lipids Health Dis..

[54] Kraus D., Yang Q., Kahn B.B. (2015). Lipid Extraction from Mouse Feces. Bio-Protoc..

[55] Diaz Marin R., Crespo-Garcia S., Wilson A.M., Sapieha P. (2019). RELi protocol: Optimization for protein extraction from white, brown and beige adipose tissues. MethodsX.

